# GDF‐15 in solid vs non‐solid treatment‐naïve malignancies

**DOI:** 10.1111/eci.13168

**Published:** 2019-09-26

**Authors:** Henrike Arfsten, Anna Cho, Claudia Freitag, Markus Raderer, Georg Goliasch, Philipp E. Bartko, Raphael Wurm, Guido Strunk, Heinz Gisslinger, Christine Marosi, Gabriela Kornek, Christoph Zielinski, Martin Hülsmann, Noemi Pavo

**Affiliations:** ^1^ Department of Internal Medicine II Division of Cardiology Medical University of Vienna Vienna Austria; ^2^ Department of Internal Medicine I Division of Oncology and Hematology Medical University of Vienna Vienna Austria; ^3^ Complexity Research Vienna Austria; ^4^ FH Campus Vienna Vienna Austria; ^5^ Technical University Dortmund Dortmund Germany

**Keywords:** biomarker, cancer, GDF‐15, inflammation, prognosis

## Abstract

**Aim:**

GDF‐15 is an established cardiovascular risk marker but is equally implicated in tumour biology. Elevated levels of GDF‐15 have indeed been observed in distinct tumour entities. This study aimed to explore the relation of GDF‐15 to other cardiac biomarkers and the general association of GDF‐15 on prognosis in an unselected cohort of treatment‐naïve cancer patients.

**Methods:**

We prospectively enrolled 555 consecutive patients at time of diagnosis of malignant disease prior receiving anticancer therapy. Plasma GDF‐15 concentrations were determined alongside other cardiac and routine laboratory markers. All‐cause mortality was defined as primary endpoint.

**Results:**

GDF‐15 levels were 338 ng/L (IQR:205‐534) for the total cohort, and values were comparable for different tumour entities except breast cancer. Metastatic disease was characterized by higher plasma GDF‐15 [435 ng/L (IQR:279‐614) vs 266 ng/L (IQR:175‐427), *P* < .001]. GDF‐15 correlated positively with inflammatory status reflected by CRP, SAA and IL‐6 [*r* = .31, *P* < .001, *r* = .23, *P* < .001 and *r* = .14, *P* = .002] and cardiac biomarkers as NT‐proBNP, hsTnT, MR‐proADM and CT‐proET‐1 [*r* = .46; *r* = .46; *r* = .59 and *r* = .50; *P* < .001 for all]. GDF‐15 was significantly associated with all‐cause mortality after multivariate adjustment [adj.HR for ln(GDF‐15) 1.78, 95%CI:1.47‐2.16, *P* < .001]. There was a significant interaction between solid and haematological malignancies with loss of association of GDF‐15 with outcome in myelodysplastic and myeloproliferative disease.

**Conclusions:**

Elevated plasma GDF‐15 is associated with progressing disease severity and poor prognosis in solid tumours of treatment‐naïve cancer patients. GDF‐15 increase is accompanied by worsening systemic inflammation and a subclinical functional impairment of different organs including the heart. GDF‐15 represents a promising target for our pathophysiologic understanding in cardio‐oncology linking conditions of both cardiac and neoplastic disease.

## 1 INTRODUCTION

Against the background of an aging population accompanied by an increasing socioeconomic burden of both cardiac and malignant disease, the main challenge of cardio‐oncology is the preservation or stabilization of cardiac function in cancer patients receiving anticancer therapy.^1^, ^2^, ^3^ To achieve this, a profound understanding of the interplay between systemic malignant disease and the myocardium is crucial. Clinicians rely on established biomarkers as N‐terminal pro‐B‐type natriuretic peptide (NT‐proBNP) or high‐sensitive TroponinT (hsTnT) as markers of cardiotoxicity. However, recent studies showed that cardiac biomarkers are already elevated in treatment‐naïve cancer patients, presumably reflecting a subclinical myocardial impairment as a reaction to the systemic disease,^4^, ^5^ making interpretation of these markers more complex. Growth differentiation factor‐15 (GDF‐15) is an emerging sensitive cardiac biomarker predicting prognosis in established cardiovascular disease but also incidence of major cardiovascular events in an apparently healthy population.^6^ As a member of the transforming growth factor‐β superfamily, GDF‐15 is equally implicated in immune regulatory processes, tissue repair, cell growth and cell survival.^7^, ^8^ Hence, GDF‐15 is also implicated in tumour genesis regulating the tumour microenvironment with a potential impact on tumour progression and invasion.^9^, ^10^ Overexpression of GDF‐15 has been reported in tissues of various types of malignant disease as colorectal, oesophageal, pancreatic, prostate, ovarian and endometrial cancers, glioma and melanoma.^11^, ^12^, ^13^, ^14^, ^15^, ^16^ Additionally, levels of circulating GDF‐15 have been related to histopathological malignant grade and metastatic progression in small studies^12^, ^15^ with the result to consider GDF‐15 a potential biomarker for tumour diagnosis and surveillance.^8^

From a cardio‐oncologic perspective, there are currently no data regarding the relationship between dysregulation of GDF‐15 and other cardiac biomarkers in cancer patients available. Furthermore, the general association of GDF‐15 on prognosis in treatment‐naïve cancer patients has not been investigated yet.

### 1.1 Rationale

The aim of this study was to determine circulating GDF‐15 levels and their association with overall survival in an unselected cohort of treatment‐naïve cancer patients as well as to assess the relationship of GDF‐15 with other established cardiovascular biomarkers to further address its role in cardio‐oncology.

## 2 MATERIALS AND METHODS

### 2.1 Study population

Consecutive patients with a primary diagnosis of cancer were prospectively enrolled at the Vienna General Hospital, a university‐affiliated tertiary care centre between April 2011 and June 2013. Eligible patients had suspected or confirmed cancer at first presentation and were excluded if they had received any prior anticancer therapy, showed clinical signs of infection or if the diagnosis of cancer could not be confirmed. Patients were classified according to tumour entity and tumour stage. Comorbidities as hypertension, cardiac diseases or diabetes mellitus and traditional risk factors as smoking status and medical therapy were recorded. Cardiac status was considered to be normal in the absence of a history of cardiac disease, electrocardiogram (ECG) abnormalities and NT‐proBNP levels <400 pg/mL. Otherwise, echocardiography was performed. Significant echocardiographic findings were defined as follows: mildly, moderately or severely reduced left or right ventricular function, moderate or severe valvular disease or diastolic dysfunction with pseudonormal or restrictive filling patterns. Abnormal cardiac status was finally defined as a history of a cardiac disease or an abnormal ECG, regardless of echocardiographic findings, or a significant echocardiographic finding in patients with NT‐proBNP levels ≥400 pg/mL. Written informed consent was obtained from all study participants. The study protocol complies with the Declaration of Helsinki and was approved by the local ethics committee of the Medical University of Vienna (EK 736/2010).

### 2.2 Laboratory analysis

Venous blood samples were obtained at first hospital presentation and routinely available laboratory parameters analysed on‐site, according to the local laboratory's standard procedures. GDF‐15, a set of other cardiac biomarkers as N‐terminal B‐type natriuretic peptide (NT‐proBNP), high‐sensitive TroponinT (hsTnT), mid‐regional pro‐adrenomedullin (MR‐proADM), mid‐regional pro‐atrial natriuretic peptide (MR‐proANP), C‐terminal pro‐endothelin‐1 (CT‐pro‐ET‐1) and copeptin as well as the inflammatory markers C‐reactive protein (CRP), serum amyloid A (SAA) and interleukin‐6 (IL‐6) were determined by specific assays.

### 2.3 Assays

GDF‐15 was measured in ethylenediaminetetraacetic acid (EDTA) plasma with a specific enzyme‐linked immunosorbent assay (ELISA) (DY957, R&D Systems) according to the manufacturer´s manual. hsTnT and NT‐proBNP measurements were performed in heparin plasma using the Elecsys System (Roche Diagnostics). MR‐proANP, MR‐proADM, CT‐proET‐1 and copeptin were measured in EDTA plasma using specific sandwich immunoassays (BRAHMS). CRP and SAA levels were determined in EDTA and heparinized plasma by means of particle enhanced immunonephelometry using the BN II System (Siemens Healthcare Diagnostics). Serum IL‐6 was detected with a specific ELISA (eBioscience).

### 2.4 Study endpoint

All‐cause mortality was chosen as the primary study endpoint. Data were obtained from the Central Office of Civil Registration Austria.

### 2.5 Statistical analysis

Continuous data were presented as median and IQR and categorical data as counts and percentages. Medians between groups were compared using the Mann‐Whitney *U* or Kruskal‐Wallis test. The Spearman Rho correlation coefficient was calculated for GDF‐15 and other variables. Cox proportional hazard regression analysis was used to evaluate the effect of GDF‐15 on all‐cause mortality in the total cohort and subgroups of cancer patients. To account for potential confounding effects, multivariate Cox regression analysis was performed adjusting for a clinical confounder model including age, gender, renal function and cardiac status and additionally for tumour entity and stage. Results are presented as HRs referring to an increase per unit of ln(GDF‐15). Interaction term analysis was performed to assess the effect of solid and haematological tumour entities on the association of GDF‐15 with outcome. For correlation assessment GDF‐15, NT‐proBNP, MR‐proANP and copeptin were entered in a logarithmic form. To assess the association of GDF‐15 levels with the primary endpoint graphically, the population was divided into tertiles and overall survival for 24 months was presented as Kaplan‐Meier curves. Groups were compared by the means of the log‐rank‐test. For all tests, two‐sided *P*‐values lower .05 were considered to indicate statistical significance. The analyses were carried out using the SPSS 22.0 software (IBM Corp).

Reporting of the study conforms to STROBE statement along with references to STROBE statement and the broader EQUATOR guidelines.^17^

## 3 RESULTS

### 3.1 Baseline characteristics

**Figure 1:**
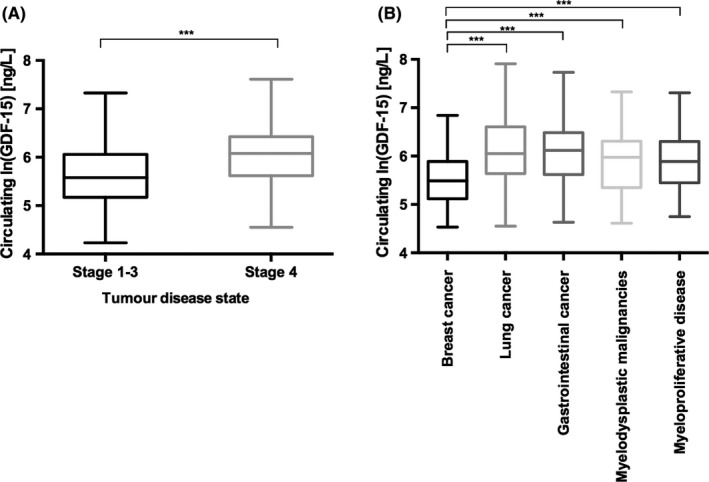
Circulating GDF‐15 levels according to disease severity and tumour site in a treatment‐naïve unselected cohort of cancer patients. GDF‐15 levels are represented as Tukey plots for A. nonmetastatic vs metastatic disease and B. different tumour sites, significant differences between groups are indicated by brackets. *** represents a statistical significance level of *P* < .001

A total of 555 consecutive patients were enrolled in this prospective cohort study. The detailed baseline characteristics of our study population are displayed in Table 1, a complete description of tumour entities is presented in Table S1. Median age was 62 (IQR 52‐71), and 41% of the patients were male. 33% of patients presented with a tumour stage 4. GDF‐15 values of the total cohort were 338 ng/L (IQR 205‐534). GDF‐15 levels were significantly lower in nonmetastatic disease compared to metastatic condition [266 ng/L (IQR 175‐427) vs 435 ng/L (IQR 279‐614), *P* < .001]. Distribution of GDF‐15 levels of the most common tumour entities is shown in Figure 1. GDF‐15 levels of the different tumour entities were comparable except for lower GDF‐15 levels in breast cancer patients. GDF‐15 levels correlated positively with age [*r* = .47, *P* < .001]. Higher GDF‐15 concentrations could be shown for patients with coronary artery disease [551 ng/L (IQR 375‐720) vs 320 ng/L (IQR 120‐516)], *P* < .001, COPD [417 ng/L (IQR 301‐635) vs 297 ng/L (IQR 189‐499), *P* < .001], CKD [542 ng/L (IQR 375‐1531) vs 323 ng/L (IQR 202‐516), *P* < .001] or diabetes [418 ng/L (IQR 271‐620) vs 325 ng/L (IQR 198‐520), *P* = .017].

**Table 1:** Baseline characteristics of treatment‐naïve patients diagnosed with cancer (n = 555)

	Treatment‐naïve cancer patients (n = 555)
Age, years (IQR)	62 (52‐71)
Male gender, n (%)	227 (41)
BMI kg/m^2^, (IQR)	25.0 (22.6‐28.4)
Comorbidities	
Known CAD, n (%)	28 (5)
Heart failure, n (%)	38 (7)
Diabetes mellitus, n (%)	43 (8)
Arterial hypertension, n (%)	250 (45)
CKD, n (%)	31 (6)
COPD, n (%)	113 (20)
Cancer disease stage^a^	
Stage 1, n (%)	96 (17)
Stage 2, n (%)	50 (9)
Stage 3, n (%)	108 (19)
Stage 4, n (%)	183 (33)
Laboratory parameters	
GFR, mL/min/1.73 m^2^ (IQR)	74.5 (63.7‐86.0)
BUN, mg/dL (IQR)	15 (12‐19)
BChE, kU/L (IQR)	7.3 (6.1‐8.4)
AST (GOT), U/L (IQR)	24 (19‐31)
ALT (GPT), U/L (IQR)	22 (16‐32)
GGT, U/L (IQR)	32 (21‐63)
Bilirubin, mg/dL (IQR)	0.6 (0.4‐0.8)
Albumin, g/L (IQR)	43.0 (40.0‐45.4)
CRP, mg/dL (IQR)	0 (0‐1)
SAA, µg/mL (IQR)	8 (4‐26)
IL‐6, pg/mL (IQR)	2 (2‐3)

Note

Continuous variables are given as medians and interquartile ranges (IQR). Counts are given as numbers and percentages.

Abbreviations: ALT, alanine transaminase; AST, aspartate transaminase; BChE, butyryl‐cholinesterase; BMI, body mass index; BUN, blood urea nitrogen; CAD, coronary artery disease; CKD, chronic kidney disease; COPD, chronic obstructive pulmonary disease; CRP, C‐reactive protein; GFR, glomerular filtration rate; GGT, gamma‐glutamyltransferase; IL‐6, interleukin 6; IQR, interquartile range; SAA, Serum Amyloid A.

^a^ Tumour stage was assessed by the respective treating oncologist and was indicated for all patients excluding those with myeloproliferative neoplasias.

### 3.2 Correlation of GDF‐15 with cardiac and inflammatory biomarkers

GDF‐15 showed a significant correlation with all cardiac biomarkers [*r* = .46, *P* < .001 for NT‐proBNP; *r* = .46, *P* < .001 for hsTnT; *r* = .47, *P* < .001 for MR‐proANP; *r* = .59, *P* < .001 for MR‐proADM; *r* = .50, *P* < .001 for CT‐proET1 and *r* = .34, *P* < .001 for copeptin], the inflammatory markers [*r* = .31, *P* < .001 for CRP, *r* = .23, *P* < .001 for SAA and *r* = .14, *P* = .002 for IL‐6] and other routine laboratory parameters generally associated with outcome in cancer patients [*r *= −.42, *P* < .001 for albumin and *r* = −.28, *P* < .001 for haemoglobin].

### 3.3 Laboratory parameters according to GDF‐15 tertiles

Table 2 shows the comparison between laboratory parameters according to GDF‐15 tertiles. The cardiac markers NT‐proBNP and hsTnT, the kidney functional parameters as glomerular filtration rate (GFR) and urea as well as the liver parameters bilirubin, γ‐glutamyltransferase (GGT), aspartate aminotransferase (AST) and albumin were significantly altered for the different GDF‐15 tertiles, with worsening of the organ‐specific parameters by increasing GDF‐15 levels.

**Table 2:** GDF‐15 levels and laboratory parameters according to tertiles in unselected treatment‐naïve cancer patients (n = 555)

	1. tertile	2. tertile	3. tertile	*P*‐value
GDF‐15, ng/L (IQR)	170 (146‐205)	338 (281‐387)	635 (534‐828)	**‐**
NT‐proBNP, pg/mL (IQR)	76 (44‐126)^a^, ^b^	139 (72‐255)^a^, §	231 (116‐729)§, b	**<.001**
hsTnT, ng/L (IQR)	3 (3‐5)^a^, ^b^	7 (3‐11)^a^, §	9 (5‐16)§, b	**<.001**
GFR, mL/min/1.73 m^2^ (IQR)	80.2 (70.4‐88.6)^a^, ^b^	73.6 (64.0‐83.0)^a^, §	66.2 (54.7‐81.1)§, b	**<.001**
BUN, mg/dL (IQR)	13 (11‐16)^a^, ^b^	16 (12‐19)^a^, §	18 (14‐23)§, b	**<.001**
Bilirubin, mg/dL (IQR)	0.6 (0.4‐0.7)^b^	0.6 (0.4‐0.8)§	0.6 (0.5‐0.9)§, b	**.029**
GGT, U/L (IQR)	26 (17‐42)^a^, ^b^	33 (24‐49)^a^, §	52 (26‐107)§, b	**<.001**
AST (GOT), U/L (IQR)	21 (18‐28)^a^, ^b^	24 (20‐30)^a^, §	28 (21‐40)§, b	**<.001**
ALT (GPT), U/L (IQR)	21 (16‐29)	23 (17‐32)	22 (15‐36)	.323
BChE, kU/L (IQR)	7.7 (6.8‐9.1)^b^	7.6 (6.4‐8.6)§	6.3 (4.9‐7.6)§, b	**<.001**
Albumin, g/L (IQR)	44.2 (42.5‐46.5)^a^, ^b^	43.3 (40.5‐45.4)^a^, §	40.6 (37.2‐43.2)§, b	**<.001**

Note

Fonts in bold indicate statistical significance (*P* < .05).

^a^ tertile1 vs tertile2.

^§^ tertile2 vs tertile3.

^b^ tertile1 vs tertile3.

### 3.4 Survival analysis

186 (34%) patients of the total cohort died during a median follow‐up of 25 (IQR 16‐32) months. Table 3 shows the association of GDF‐15 with outcome for the total cohort as well as the most common tumour entity subgroups. GDF‐15 was significantly associated with all‐cause mortality in the univariate analysis for the total cohort [crude HR for ln(GDF‐15)2.08, 95% CI:1.77‐2.43, *P* < .001]. This association remained significant after adjustment for age, gender, kidney function, cardiac status, tumour entity and tumour stage [adjusted HR for ln(GDF‐15)1.78, 95% CI:1.47‐2.16, *P* < .001]. In the subgroup analysis, GDF‐15 was similarly significantly associated with outcome for solid tumours as breast cancer, lung cancer or gastrointestinal cancer; however, no association with outcome could be shown for haematological cancers as myelodysplastic or myeloproliferative diseases. Association of GDF‐15 with outcome for the total cohort and in solid tumours was also independent from NT‐proBNP and hsTnT levels (data not shown). Interaction term analysis confirmed the significant effect of solid vs haematological tumour entities on the association of GDF‐15 with outcome (*P* = .024).

**Table 3:** Association of GDF‐15 levels with all‐cause mortality in unselected treatment‐naïve cancer patients according to tumour site (n = 555)

	Crude HR	*P*‐value	Adj. HR	*P*‐value
Total cohort (n = 555)	2.08 (1.77‐2.43)	**<.001**	1.78 (1.47‐2.16)^a^	**<.001**
Breast cancer (n = 146)	6.27 (3.26‐12.05)	**<.001**	5.47 (2.66‐11.24)^b^	**<.001**
Lung cancer (n = 61)	1.66 (1.17‐2.37)	**.005**	1.90 (1.30‐2.77)^b^	**<.001**
Gastrointestinal cancer (n = 67)	1.62 (1.17‐2.25)	**.004**	1.91 (1.33‐2.74)^b^	**<.001**
Myelodysplastic neoplasia (n = 68)	1.25 (0.71‐2.21)	.443	1.29 (0.73‐2.25)^b^	.381
Myeloproliferative disease (n = 99)	1.42 (0.53‐3.80)	.480	1.17 (0.35‐3.91)^b^	.802

Note

HR refers to an increase per unit of ln(GDF‐15).

Fonts in bold indicate statistical significance (*P* < .05).

^a^ Adjusted for age, gender, kidney function (GFR), cardiac status, tumour entity and stage.

^b^ Adjusted for age and kidney function.

### 3.5 Kaplan‐Meier curves

**Figure 2:**
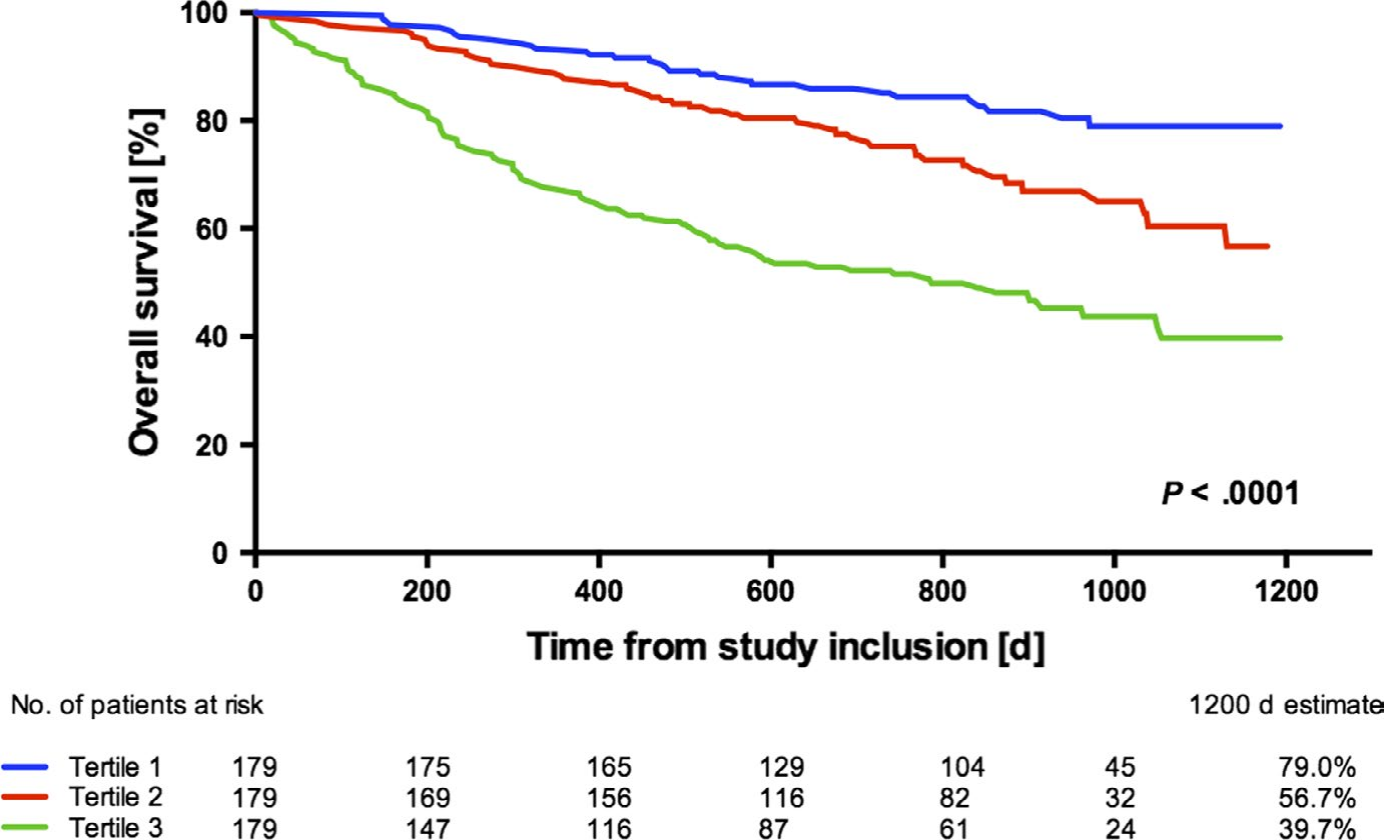
Overall survival rates for treatment‐naïve cancer patients (n = 555) according to tertiles of GDF‐15 (*P* < .001 between all groups, log‐rank test)

Kaplan‐Meier curves and log‐rank analysis shown in Figure 2 confirmed the high discriminatory power of GDF‐15 on overall survival for treatment‐naïve cancer patients. The 12‐ and 24‐month estimates were 93.3% and 85.9% in the lower, 88.3% and 76.0% in the mid and 67.6% and 52.3% in the upper tertile (*P* < .001 between all groups). Kaplan‐Meier curves and log‐rank analysis for different solid tumours and haematological malignancies according to GDF‐15 tertiles of the respective subgroups are displayed in Figure S1.

## 4 DISCUSSION

This is the first study investigating the role of GDF‐15 within the scope of the interdisciplinary field of cardio‐oncology. GDF‐15 was associated with all‐cause mortality in the total cohort, whereas in the subgroup analysis, interestingly, association with outcome remained significant only for solid tumours but not for myelodysplastic or myeloproliferative disease, indicating different disease‐related mechanisms of the protein. Circulating GDF‐15 levels correlated significantly with other established cardiovascular biomarkers and inflammatory status in treatment‐naïve cancer patients in line with the proposed presence of a subclinical myocardial involvement with worsening disease severity.

### 4.1 GDF‐15 levels in comparison

Circulating GDF‐15 levels have been measured under different pathophysiological conditions with different analytical methods for research use. Using a sandwich ELISA of the same manufacturer, studies have reported median plasma GDF‐15 levels of 309 ng/L (IQR 275‐411) for healthy individuals and 427 ng/L (IQR 344‐626) in obese patients,^18^ or median levels of 1097 ng/L and 5753 ng/L for healthy and critically ill patients.^19^ Regarding malignant disease, GDF‐15 levels were determined with a mean value of 746 ng/L in controls vs 1075 ng/L in colorectal cancer patients.^20^ GDF‐15 levels within this study correspond well to these data. GDF‐15 levels appear somewhat lower than plasma concentrations of apparently healthy individuals determined by IRMA or the Roche assay in other studies,^21^, ^22^ which may be based on the nature of a manual technique or that the ELISA method seems to results in lowest GDF‐15 values compared to IRMA or the Elecsys® measurements.^23^ Altogether a very good correlation was shown for the ELISA method with the just recently available first clinically approved Elecsys® assay (Roche Diagnostics).^23^

### 4.2 GDF‐15 in malignant disease

GDF‐15 or macrophage inhibitory cytokine‐1 (MIC‐1), a member of the transforming growth factor‐β superfamily cytokines, is implicated in cell growth and homoeostasis and thereby potentially modulating tumour progression and invasiveness.^8^ Although under physiological conditions GDF‐15 is generally regarded to exert anti‐inflammatory and anti‐tumourigenic effects, microarray data reveal that GDF‐15 is a protein with a high level of tumour‐associated expression.^24^ Overexpression of GDF‐15 has been confirmed in various types of malignant disease.^9^, ^11^, ^12^, ^13^, ^16^ GDF‐15 is also the only known secreted p53‐regulated cytokine reflecting p53 activation,^10^ anticipating a promising role as biomarker. A strong association between circulating levels of GDF‐15 and the histopathological grade of malignancy, tumour mass and metastatic progression has been documented for colorectal, pancreatic, prostate and oral squamous cancer in mostly small studies.^9^, ^14^, ^15^, ^16^, ^25^ As a consequence, GDF‐15 has been discussed as a potential biomarker for tumour diagnosis and surveillance.^8^ However, larger studies investigating the prognostic association of GDF‐15 in an unselected cohort of especially treatment‐naïve cancer patients have not been conducted.

In our cohort of treatment‐naïve cancer patients, circulating GDF‐15 levels were comparable for most common tumour entities, except for slightly lower values in breast cancer. One study comparing GDF‐15 levels of pancreatic cancer patients with several other tumour entities similarly revealed lower concentrations of GDF‐15 for breast cancer patients,^25^ albeit in vitro data suggest a participation of GDF‐15 in malignant progression of breast cancer cell lines.^26^ Importantly, we found the highest crude hazard ratios for GDF‐15 in breast cancer patients in our analysis. Metastatic disease was characterized by higher circulating GDF‐15 levels compared to less advanced stages, in line with the reported data. GDF‐15 was associated with all‐cause mortality in the total unselected cohort encompassing different types of malignant disease. Interestingly, further analysis revealed a significant interaction for the subgroups of solid tumours and haematological malignancies, whereas GDF‐15 was no longer associated with survival for patients suffering from myeloproliferative or myelodysplastic disease.

Although GDF‐15 has been extensively investigated in solid tumours, there are only scarce data regarding haematological malignancies. For multiple myeloma, an increased survival of stroma‐dependent multiple myeloma cells at high levels of GDF‐15 linked to worse event‐free survival as well as an enhanced tumour‐initiating and renewal potential have been described.^14^, ^27^ Moreover, an in vitro study reported GDF‐15 mediated protection of AML cell lines.^28^ Our data cannot confirm a general prognostic significance of GDF‐15 for haematological malignancies, yet a specific association of GDF‐15 in multiple myeloma cannot be excluded. It seems that GDF‐15 overexpression and secretion is more characteristic for the solid tumour entities, indicating a different role of GDF‐15 for cancerous cell lines of haematological malignancies.

### 4.3 GDF‐15 and inflammation

GDF‐15 was originally identified simultaneously by different groups and approaches in the late 1990s,^29^ one of them by searching for genes related to macrophage activation.^30^ GDF‐15 is upregulated by a variety of inflammatory stimuli and might serve as an autocrine regulator of macrophage activation.^29^ In apparently healthy individuals, GDF‐15 is widely but only weakly expressed in most tissues, suggesting its implication in basic cellular functions.^29^ However, being a key secretory cytokine responding to multiple cellular stressors, it is less surprising that an increase in circulating GDF‐15 levels generally accompanies pathophysiologic conditions as acute injury, inflammation or cancer. Although malignant diseases strongly rely on genetic and environmental factors, there is increasing evidence that the host inflammatory response plays an important role in the development and progression of cancer. Elevated circulating GDF‐15 levels were not only associated with future cancer mortality in established disease but also for a cancer‐free population at baseline,^31^ and higher GDF‐15 levels were associated with an increased cancer incidence in a patient cohort with diabetes.^32^ Here, GDF‐15 increase could reflect an unfavourable pro‐inflammatory state setting the stage for the development of malignant disease. For manifest malignant disease, inflammation based scores using more classic inflammatory markers as CRP or albumin indeed have been shown to have prognostic value, independent of tumour entity or stage.^33^

To underline the pathophysiologic relation of GDF‐15 with inflammatory state, we have determined the inflammatory markers CRP, SAA, IL‐6 as well as albumin and could show that all parameters showed a significant correlation with GDF‐15.

### 4.4 GDF‐15 in the field of cardio‐oncology

Expression of GDF‐15 is upregulated in atherosclerotic artery wall and elevated circulating levels reflect endothelial activation and vascular inflammation.^6^ Cardiomyocytes produce GDF‐15 at very low levels; however, ischaemic injury rapidly upregulates GDF‐15 in the affected area.^34^ GDF‐15 has been described as a risk factor in patients suffering from various cardiovascular diseases as coronary artery disease and myocardial infarction, heart failure, or atrial fibrillation.^35^, ^36^, ^37^, ^38^ Even in apparently healthy individuals, GDF‐15 levels may predict risk of future myocardial infarction or cardiovascular death,^31^ as that GDF‐15 can now be regarded as an established cardiac biomarker.

Given an aging population, an increasing incidence of malignant and cardiac diseases and their interdependency, cardio‐oncology has emerged as an essential interdisciplinary field. One main challenge is predicting cardiotoxic reactions of anticancer therapies and the surveillance and preservation of cardiac function of cancer patients.^1^, ^39^ However, recent studies showed elevated cardiac biomarkers in cancer patients without manifest cardiac disease, assumedly reflecting a subclinical myocardial impairment as a reaction to systemic disease.^4^, ^5^ A more recent analysis of this treatment‐naïve cancer cohort revealed an increase in functional as well as morphological cardiac biomarkers as NT‐proBNP, MR‐proANP, MR‐proADM, CT‐proET‐1, copeptin and hsTnT and that all markers are associated with worse prognosis.^40^, ^41^ A similar involvement seems to apply for other organ systems as already described for the liver with elevated functional parameters in the setting of malignant disease without direct organ damage.^42^

In this study, we show that GDF‐15 displays a strong correlation with the above mentioned markers and is similarly associated with prognosis. Kidney and liver functional parameters equally rise with increasing GDF‐15 tertiles. GDF‐15 may therefore represent another sensitive link between malignant disease and the heart with a potentially additional diagnostic value for future exploitation.

## 5 LIMITATIONS

One potential limitation of this study is the unselective nature of patient enrolment including various types of cancer. Nevertheless, we intended to investigate the alterations of GDF‐15 as a general phenomenon in cancer, without focusing on distinct tumour entities. Further studies might reveal more differences between various types of cancer. Laboratory measurements have been performed only at a single time point prior to initiation of anticancer therapy, and studies with serial measurements throughout disease progression might provide additional insights.

These data cannot prove the pathophysiologic concept that malignant disease directly affects the myocardium. An increase in cardiac markers in cancer patients might be based on conditions which promote heart disease and cancer in parallel. On the other hand, cancer is an inflammatory disease and inflammation is a trigger for the development and progression of heart disease. Two landmark trials have proved this concept: obviously healthy patients with elevated CRP profit from an immune‐modulatory therapy with rosuvastatin^43^ and, more recently, it has been shown that a specific anti‐inflammatory treatment with canakinumab reduces cardiovascular events.^44^

## 6 CONCLUSIONS

Elevated plasma GDF‐15 is associated with progressing disease severity and poor prognosis in solid tumours of treatment‐naïve cancer patients. GDF‐15 increase is accompanied by worsening systemic inflammation and a subclinical functional impairment of different organs including the heart. GDF‐15 represents a promising target for our pathophysiologic understanding in cardio‐oncology linking conditions of both cardiac and neoplastic disease.

## CONFLICT OF INTEREST

None.

## FUNDING INFORMATION

This study was supported by an unrestricted grant of Thermo Fisher.
