# Supplementary figures and images for: GDF‐15 in solid vs non‐solid treatment‐naïve malignancies
Source: Eur J Clin Invest. 2019 Sep 26;49(11):e13168. doi: 10.1111/eci.13168 (PMC6899906; doi:10.1111/eci.13168)

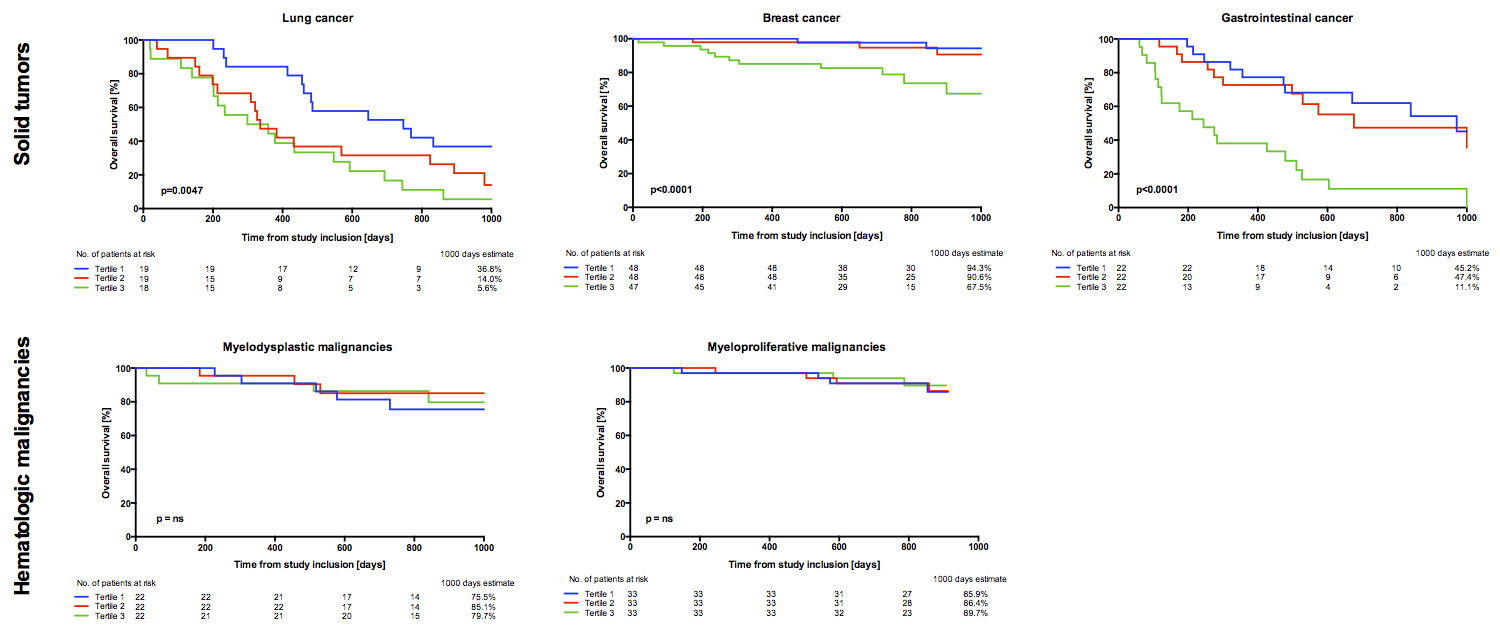

Supplement: Supplementary file 1 [file ECI-49-na-s001.tiff]
