# Supplementary material for: GDF‐15 in solid vs non‐solid treatment‐naïve malignancies
Source: Eur J Clin Invest. 2019 Sep 26;49(11):e13168. doi: 10.1111/eci.13168 (PMC6899906; doi:10.1111/eci.13168)
Supplement: Supplementary file 2 [file ECI-49-na-s002.docx]

**Supplemental Table S1.** **Tumor entities of treatment-naïve cancer patients (n=555).** Counts are given as numbers and percentages.

|  | **Cancer patients (n=555)** | |
| --- | --- | --- |
| **Tumor entity** |  | |
| Lung cancer, n (%) | | 61 (11.0) |
| Breast cancer, n (%) | | 146 (26.3) |
| Brain tumor, n (%) | | 23 (4.1) |
| ENT-tumor, n (%) | | 33 (5.9) |
| Gastrointestinal tumors, n (%) | | 67 (12.1) |
| Myelodysplastic malignancies, n (%) | | 68 (12.3) |
| Myeloproliferative neoplasias, n (%) | | 99 (17.8) |
| Esophageal cancer, n (%) | | 11 (2.0) |
| Testicular cancer, n (%) | | 2 (0.4) |
| Neuroendocrine tumor, n (%) | | 11 (2.0) |
| Sarcoma, n (%) | | 9 (1.6) |
| Mesothelioma, n (%) | | 3 (0.6) |
| Prostate cancer, n (%) | | 2 (0.4) |
| Renal cell carcinoma, n (%) | | 4 (0.7) |
| Thymoma, n (%) | | 1 (0.2) |
| Skin cancer, n (%) | | 2 (0.4) |
| Urogenital tumors, n (%) | | 2 (0.4) |
| Oral cancer, n (%) | | 1 (0.2) |
| Other, n (%) | | 10 (1.8) |

ENT – ear, nose, throat; Myelodysplastic malignancies – haematological malignancies with abnormal differentiation of myeloid or lymphoid cell lines (e.g. AML, ALL, lymphomas, multiple myeloma); Myeloproliferative neoplasias – haematological neoplasias with normal cell differentiation (e.g. essential thrombocytosis, polycythemia vera, myelofibrosis).
